# Death by suicide during COVID-19 infection: Two case reports

**DOI:** 10.5339/qmj.2023.12

**Published:** 2023-07-28

**Authors:** Majid Alabdulla, Rajeev Kumar

**Affiliations:** ^1^Consultation-Liaison Psychiatry Unit, Hamad Medical Corporation, Doha, Qatar E-mail: Rkumar5@hamad.qa ORCID: https://orcid.org/0000-0002-7524-4742; ^2^College of Medicine, Qatar University, Doha, Qatar

**Keywords:** Suicide, COVID-19, mental health, psychosocial, suicidal behavior

## Abstract

A range of psychiatric disorders has been recognized with coronavirus disease 2019 (COVID-19) infection, including acute stress, anxiety, depression, suicidal behavior, and post-traumatic stress disorder. Among those, the most worrying is death by suicide, which has been associated with COVID-19-related psychiatric disorders and psychosocial stressors. We report the first two cases of death by suicide, unlikely due to any current psychiatric disorders, while undergoing treatment in two inpatient facilities designated for COVID-19 patients. Case 1 was a 40-year-old man who presented to the emergency department with symptoms of a viral infection. This led to the diagnosis of COVID-19. While undergoing treatment in an inpatient facility, 3 weeks later, he died by hanging. Case 2 was a 25-year-old man with COVID-19-related upper respiratory tract symptoms and a possible undiagnosed pre-existing anxiety disorder. While undergoing treatment in a medical unit of a COVID-19-designated hospital, a week after the diagnosis of COVID-19, the patient died after jumping off the multistory hospital building. In both cases, there had been a diagnosis of COVID-19, and treatment was provided within an inpatient facility. Both patients were unvaccinated and had no evidence of a current psychiatric disorder or any warning signs of suicidal intent. Death by suicide can occur in COVID-19 patients without any warning signs of a psychiatric disorder or evidence of any apparent distress. Therefore, even without a diagnosable mental disorder, clinicians should still be vigilant about potential suicidal risk in patients with COVID-19 infection.

## Introduction

Coronavirus disease 2019 (COVID-19) is a highly contagious infectious disease caused by Severe Acute Respiratory Syndrome Coronavirus-2 (SARS-CoV-2). It became a pandemic with major global health concerns. The change in this global situation challenged governments and health systems worldwide with high and growing mortality rates and variations in several countries, and the impact was profound.^1^ In addition to the physical morbidity and mortality associated with this disease, countries worldwide took unprecedented measures to control the spread of the virus. These measures included the closure of international borders; implementation of total flying bans; asking the public to follow physical distancing measures such as isolation and quarantine; the wearing of face coverings; the closure of schools, universities, and workplaces; and the separation from family and friends, all of which impacted on people’s lives emotionally and economically.

In a rapid review of the psychological impact of quarantine, the authors reported significant adverse effects that included post-traumatic stress symptoms, confusion, and anger.^2^ Furthermore, they showed evidence that these effects correlated with prolonged quarantine, infection fears, frustration, boredom, inadequate supplies, inadequate information, financial loss, and stigma. There have been several reports about mental health concerns related to COVID-19 since its outbreak in December 2019 in Wuhan, Hubei Province, China. An online survey of 1,219 patients showed that approximately 53.8% of COVID-19 patients had a moderate to severe psychological impact on them.^3^ With the increasing burden of mental health problems such as severe distress, anxiety, and depression, an increased risk of suicide is inevitable. Historically, this has been the case in the United States during the 1918–1919 influenza pandemic^4^ and in Hong Kong during the 2003 severe acute respiratory syndrome (SARS) epidemic.^5^ We recently reported a prevalence of 24.9% of suicidal behavior among all patients presenting to the emergency room with acute psychiatric disorders during the peri-COVID-19 period.^6^ To further set the context, Qatar has a total population of approximately 2.8 million, of which 98.5% are younger individuals.^7^ Of note, Qatar has an expatriate population of approximately 89%, of which about half are manual and craft workers accommodated in high-density areas housed in shared facilities.^8^ In a recent review, it has been reported that several factors could put Qatar’s residents at increased risk of COVID-19 transmission, including attending gatherings during religious holidays, having a high-capacity international airport, having high-density accommodations, and their work surroundings.^9^ In an important study on a 1-year review of COVID-19 status in the Arab world, it was shown that Qatar, Bahrain, and Lebanon showed the highest number of recovered, confirmed, and deaths per million populations, but the younger Arab population contributed to fewer deaths compared to other top-most affected countries worldwide.^10^ Importantly, in a study on participants registered at the Primary Health Care Corporation, which is the main public primary healthcare provider in Qatar and operates in 27 health centers with about 1.4 million registered patients, a sharp increase in the COVID-19 positivity rate was noted from March to May 2020, with the highest rate of 37.5% of those tested in May.^11^ In the same study, it was noted that the younger Asian male population had a positivity rate of 15.7% of the total number of people tested with the highest risk among Asians than those originally from the Middle East and North Africa (OR 1.29; 95% CI 1.27–1.32). In Qatar, the COVID-19 drive-through screening process started on March 2, 2020, with the first case documented on February 29, 2020, among repatriated citizens out in quarantine.^12^ It is important to note that Qatar has an excellent health system with modern healthcare facilities.^13^ During the pandemic, the health system responded quickly to increase its capacity by turning five of its hospitals into COVID-19 facilities and keeping the other hospitals as non-COVID-19 facilities.^14,15^ A COVID-19 frontline healthcare facility in Qatar called Hazm Mebaireek General Hospital increased its ICU bed capacity from 7 to 250 beds with full critical care support.^16^ Of note, three temporary tents fully supported by oxygen supply units were erected as a key triage hub for cases across Doha to reduce the unnecessary admission rate.

There have been several case reports of deaths by suicide since the start of COVID-19. In most cases, an exact reason was mentioned, such as stress, financial strain, inter-personal conflict, work stress, uncertainties about potential treatment and access to treatment, stress from not being able to meet educational needs, the onset of a psychotic episode, and fear and stigma from COVID-19.^17–25^ In a survey conducted in Qatar between July and August 2020, it was reported that 37% of subjects had depression and 19.3% had anxiety, and subjects with anxiety were more likely to have lower wagers, whereas subjects with depression were more likely to have high wagers.^26^ The existing literature supports the view that several psychosocial and clinical factors can increase a person’s risk of suicide.^4,5,17^ Here we report two cases of death by suicide that occurred while patients were undergoing treatment in two of our medical inpatient facilities designated for the treatment of COVID-19, without indication of any psychopathology or warning signs of an impending suicide.

## Case Report

### Case 1

A 40-year-old man presented to the emergency department (ED) on April 29, 2020, with complaints of nasal congestion, difficulty breathing, and a dry, irritating cough for the previous 15 days. He denied any history of recent travel or contact with COVID-19 patients. His tympanic temperature was recorded at 36.1°C, his respiratory rate was 18 beats per minute, and his blood pressure was 135/99 mm of mercury. His systems examination was unremarkable. He was fully alert and cognitively intact. He was diagnosed with allergic rhinitis, started on dextromethorphan cough suppressant and levocetirizine, and was discharged home with 1 day of sick leave. A diagnosis of COVID-19 infection was not considered, so a polymerase chain reaction (PCR) test was not offered. He was unvaccinated.

The next day, he presented to the ED with similar symptoms. Besides, he appeared restless and had more difficulty breathing. A chest X-ray was reported as normal. Additionally, he reported that the tent where he was staying collapsed, and he sustained some minor scalp lacerations. It was unclear from the notes why he was admitted to the tent before his second presentation to the ED. A fully medically equipped tent was part of the extended care of patients with mild COVID-19 symptoms who might need observation but did not require hospital or intensive care unit admission. He also complained of a headache. A CT brain scan was reported normal on that day. A COVID-19 PCR test was done on this occasion, and the result was positive. The next day, he appeared clinically stable without any distress. He was started on daily hydroxychloroquine 400 mg and azithromycin 500 mg and was transferred to a COVID-19-designated medical inpatient facility for further management. He had no history of any mental health issues.

After 3 weeks, while in the facility, he was found dead in his room, having hanged himself using his bed sheet. During that 3-week period, there was no documentation in his file of any evidence of a disturbance in his mental state or thoughts of ending his life.

### Case 2

A 25-year-old man was brought to the ED on May 13, 2020, with complaints of fever, sore throat, headache, and body aches for 3 days. The patient underwent a COVID-19 PCR test and awaited the results. However, as he had only minor symptoms, he was given symptomatic treatment and sent home. The next day, his COVID-19 PCR was reported as positive. Of note, he was unvaccinated. After 2 days, he presented back to the ED with a fever, and he was soon transferred to the hospital’s medical unit dedicated to the treatment of COVID-19 patients. The physical examination was normal, and he was cognitively intact. He was started on hydroxychloroquine 400 mg daily and azithromycin 1000 mg. He appeared calm and was compliant with his medication. The plan was to transfer him to another dedicated inpatient facility when a bed became available. The transfer decision was based on his improvement in clinical status, and his case was categorized as low acuity.

On the night of the admission, the patient informed the nurse that he was nauseous and wanted medications to alleviate it. He was given a 10 mg tablet of metoclopramide before returning to bed. He returned to the nursing station approximately 2 hours later to ask for a pair of socks. He also asked about the transfer to the new quarantine facility. The nurse reassured him and addressed his concerns before he, once again, returned to his room, informing the nurse that he could not fall asleep and wanted an injection to help treat his insomnia, but that was not acted upon.

The following evening, the patient insisted on his discharge, despite his pending transfer to another quarantine facility. This time, the healthcare team found it more challenging to communicate with the patient, and he was reluctant to return to his room, but eventually, he did. It was noted by the nurse during her rounds four hours later that the patient was calm and not displaying any signs of agitation or frustration. While the patient was not reported missing by anyone, a few hours later, he was found dead on the hospital floor. On examination of the body, the patient sustained multiple injuries after jumping off the rooftop of the hospital building.

There were no reports of suicidal ideation during his stay. However, when we reviewed his notes, the previous hospital record revealed multiple presentations to the ED with complaints of abdominal pain, generalized body aches, dizziness, and headaches. A physician noted him having anxiety and irritable bowel syndrome in the past, but no formal psychiatric diagnosis was found in the notes.

## Discussion

We report two cases of death by suicide. Both were male, young, and Asian expatriates undergoing treatment for COVID-19 in an inpatient facility designated for treating patients with COVID-19. In the first case, death happened about 3 weeks after he tested positive for COVID-19, and in the second case, death occurred a week after he tested positive. Besides, in both cases, there were no overt psychiatric disorders or any obvious warning signs of an impending suicide at the time of admission. However, in hindsight, in the first case, there was an incident in which his living tent collapsed before admission, which could have been a traumatic event. In the second case, his previous case notes revealed evidence of a pre-existing anxiety disorder, and his restlessness and insomnia could have been signs of an exacerbation of an anxiety disorder. Both incidents happened during the emergence of the first wave of the pandemic, and understandably, there was heightened anxiety among all residents during this period. It is important to note that vaccination was not available during this period. We believe these two are the first reported cases of death by suicide in inpatient facilities designated for the protection and treatment of COVID-19 patients in Qatar in a short span of being diagnosed with the disease.

In a recent systematic review and meta-analysis, the pooled prevalence of suicidal ideation was 20.4% and suicide attempts were 11.4% among psychiatric patients during COVID-19.^27^ Further, several studies did not see an increased rate of suicidal behavior immediately after the COVID-19 pandemic.^6,28,29^ In the Spanish study that compared the monthly crude incidence of suicide-related thoughts and behaviors (STBs) and suicide mortality rates of 2020 with those of 2019, STBs in 2020 decreased by an average of 6.3%, and the authors suggested that restrictive measures implemented in response to the COVID-19 pandemic might have discouraged people from contacting mental health services.^29^ The impact of COVID-19 on people with mental illnesses, whether chronic or new-onset mental health disorders, is enormous because of the occurrence of fear, social isolation, and physical distancing.^30^ Other general immediate, medium, and long-term risk factors for suicide are loss of employment and financial stressors,^31^ alcohol use and domestic violence,^32^ and most importantly, social isolation and loneliness.^33^

Based on trend analysis of adjusted suicide rates and unemployment rates since 1900 in the United States, Canada, and Australia, it has been shown that economic depression, high unemployment rates, social isolation, and male gender are all risk factors for increased suicide risk.^34^ Although any of the factors discussed above were possible in our cases, we cannot be sure if those factors were present, as a psychiatric assessment was not considered necessary in both cases.

Although acute stress, anxiety, depression, suicidal behavior, and post-traumatic stress disorder are most likely due to the psychosocial impact of the infection, the cognitive, behavioral, and neuropsychiatric manifestations of COVID-19 infection are possibly also due to the neurotropic and neurotoxic effects of the coronaviruses.^35^ Again, we do not have any evidence to support the hypothesis that our patients had any COVID-19-related brain issues. Both patients did not show evidence of altered sensorium or cognitive impairment.

As discussed earlier, the psychosocial impact of COVID-19 might be speculated as the suicidal cause in both cases. Both were young Asian expatriates with lower-income wages. Of note, both patients died during the peak of the first wave of the pandemic, and it has been reported that anxiety and depression were prominent during this period.^26^ In Qatar, the first COVID-19 vaccination was rolled out in December 2020.^36^ Interestingly, it has been shown that there was a downward trend in the level of depression and anxiety symptoms after the first wave of COVID-19 in Qatar.^37^

Both cases were treated with azithromycin and hydroxychloroquine. The reported psychiatric side effects of azithromycin include psychotic depression and catatonia,^38^ and hydroxychloroquine include severe behavioral toxicity, such as psychosis, delirium, agitation, suicidality, personality changes, depression, and sleep disturbances.^39^ In our two cases, none of the above medication side effects were reported.

Regarding whether the two deaths were preventable, we believe both happened abruptly without any warning signs and could not have been prevented. It is also important to remember that the suicide prevention literature demonstrates that all the tools and techniques available are unfortunately unhelpful in predicting who will eventually attempt or commit suicide.^40^ Approximately 60% or more of patients who denied suicidal ideation died by suicide within 2 days of this denial.^41,42^ Despite this poor prediction of prevention, good clinical practice still involves engaging with the patients continuously, abating any distress, and sorting out any modifiable risk factors. We also suggest that all patients with COVID-19 should be regularly screened for any underlying mental health issues, even if they do not volunteer such experiences, particularly anxiety, depression, distress, and suicidal thoughts. Furthermore, at the community level, several recommendations have been made by others to prevent suicide. These include risk identification strategies, easy and immediate access to mental health services, financial support, de-stigmatization, and clear public health policies and protocols. ^43–45^

The main strength of our two case reports is that we could observe both cases in an inpatient facility, and none had any precise psychiatric diagnosis to account for the death. One limitation was that we did not use any screening tools for the identification of mental disorders and the risk of suicide.

## Conclusion

Death by suicide can occur in COVID-19 patients without any warning signs of psychiatric disorders such as anxiety, depression, psychosis, post-traumatic stress disorder, or evidence of any apparent general distress. Therefore, even without a diagnosable mental disorder, clinicians should still be vigilant about potential suicidal risk in patients with COVID-19 infection. Furthermore, we believe that the routine use of a screening tool for risk assessment of suicidal behavior should be mandatory in all cases.

### Acknowledgment

We acknowledge Mr. Iain Tulley, Chief Executive Officer of Mental Health Services at the Hamad Medical Corporation, for his helpful comments on the manuscript and confirm that we have not provided any personal or sensitive information.

### Authors contributions

MA collected the patients’ details and reviewed the manuscript. RK wrote the manuscript. Both authors contributed to the preparation of the manuscript and approved the final version.

### Availability of data and materials

We will make the complete electronic data for the two cases available upon request.

### Competing interests

We declare that we have no competing interests.

### Ethics approval and consent to participate

The study was approved by the Institutional Review Board of Hamad Medical Corporation (MRC-04-22-693). We confirm that the data provided is fully anonymized.

### Funding

This study received no specific grant from any funding agency in the public, commercial, or not-for-profit sectors.
